# Open and/or laparoscopic one-stage resections of primary colorectal cancer and synchronous liver metastases: An observational study

**DOI:** 10.1097/MD.0000000000025205

**Published:** 2021-03-19

**Authors:** Hiroaki Nozawa, Takeaki Ishizawa, Hideo Yasunaga, Hiroaki Ishii, Hirofumi Sonoda, Shigenobu Emoto, Koji Murono, Kazuhito Sasaki, Kazushige Kawai, Nobuhisa Akamatsu, Junichi Kaneko, Junichi Arita, Kiyoshi Hasegawa, Soichiro Ishihara

**Affiliations:** a Department of Surgical Oncology; b Hepato-Biliary-Pancreatic Surgery Division, Department of Surgery, Graduate School of Medicine; c Department of Clinical Epidemiology and Health Economics, School of Public Health, The University of Tokyo, Tokyo, Japan.

**Keywords:** colorectal liver metastasis, laparoscopic surgery, one-stage resections, outcomes

## Abstract

One-stage resections of primary colorectal cancer and liver metastases have been reported to be feasible and safe. Minimally invasive approaches have become more common for both colorectal and hepatic surgeries. This study aimed to investigate outcomes of these combined surgical procedures among different approaches.

We retrospectively analyzed patients diagnosed as having primary colorectal cancer with synchronous liver metastases and who underwent 1-stage primary resection and hepatectomy with curative intent in our hospital. According to the surgical approach for the primary tumor and hepatic lesions, namely open laparotomy (Op) or laparoscopic approach (Lap), patients were classified into Op-Op, Lap-Op (laparoscopic colorectal resection plus open hepatectomy), and Lap-Lap groups, respectively. Clinicopathological factors were reviewed, and short- and long-term outcomes were compared among the groups.

The Op-Op, Lap-Op, and Lap-Lap groups comprised 36, 18, and 17 patients, respectively. The superior/posterior hepatic segments were more frequently resected via an open approach. There was no laparoscopic major hepatectomy. The median volume of intraoperative blood loss was smaller in the Lap-Lap and Lap-Op groups (290 and 270 mL) than in the Op-Op group (575 mL, *P* = .008). The hospital stay after surgery was shorter in the Lap-Lap and Lap-Op groups (median: 17 days and 15 days, vs 19 days for the Op-Op group, *P* = .033). The postoperative complication rates and survivals were similar among the groups.

Application of laparoscopy to 1-stage resections of primary colorectal cancer and liver metastases may offer advantages of enhanced recovery from surgical treatment, given appropriate patient selection.

## 1 Introduction

Colorectal cancer (CRC) is one of the most common malignancies and causes of cancer death worldwide.^[1]^ On initial presentation, 15% to 20% of CRC patients have distant metastases with hepatic involvement being the most frequent.^[2–4]^ Surgery is the mainstay of treatment for stage IV CRC patients when resectable.^[5]^ With improvements in perioperative management, colorectal and hepatic resections can be safely performed simultaneously.^[6,7]^ One-stage resections of CRC and liver metastases are considered to relieve patients’ psychological stress, and save healthcare resources provided that the operative risk is not increased.^[3]^ The number of patients eligible for these aggressive treatments used to be limited; however, advances in modern chemotherapy have rendered formerly unresectable metastases resectable in a subset of advanced CRC patients,^[8,9]^ and thus more CRC patients with liver metastases have become eligible for surgical intervention.

With minimum invasiveness and equivalent outcomes to open approaches demonstrated by past randomized controlled trials (RCTs),^[10–13]^ laparoscopic colectomy has become widely adopted. Even for rectal cancer, the laparoscopic approach is gaining acceptance due to several advantages.^[14–17]^ The role of laparoscopic hepatectomy has also expanded.^[18–20]^ Moreover, an RCT named “OSLO-COMET” showed advantages of laparoscopic parenchymal-sparing hepatectomy for metastases from CRC while preserving oncological safety, over open surgery.^[21]^ Meanwhile, several studies have reported the feasibility of simultaneous resections of primary CRC and hepatic metastases via a laparoscopic approach.^[22,23]^ Application of the laparoscopic approach to both lesions will not only reduce the length of incisions but may provide further substantial advantages to these patients. Since only application of laparoscopy to colorectal surgery was associated with good short-term outcomes, its benefits facilitated 1-stage resections in patients with colorectal liver metastasis.^[24,25]^ However, only a few studies have compared the surgical outcomes via a totally laparoscopic approach with those via totally open surgery.^[26–29]^

The aim of the present study was to characterize patients with colorectal liver metastasis who underwent 1-stage laparoscopic resections of all lesions, and to compare the surgical outcomes among open, laparoscopic, and mixed approaches to address whether partial or total application of laparoscopic approaches provides benefits for these patients.

## 2 Methods

### 2.1 Surgery

In our hospital, primary CRC and synchronous liver metastases have routinely been resected simultaneously.^[30]^ Standard laparoscopic colorectal resection was introduced for early tumors around 2003, and we gradually expanded use of the laparoscopic approach for advanced CRC after 2012. Laparoscopic minor hepatectomy such as lateral segmentectomy and local resection was introduced in 2008, and its indications have gradually been expanded to other hepatectomies. Surgical approaches were discussed at a multidisciplinary team meeting. Two different teams of experienced surgeons performed open or laparoscopic resections of primary CRC and liver metastases respectively.

For open surgery, a midline incision and/or a J-shaped incision was made to patients in the supine or lithotomy position.

For colon cancer, tumor-bearing segment was mobilized and central vessels were identified and ligated. For rectal cancer, the inferior mesenteric artery was ligated at the root or at the level distal to the branching of the left colic artery. Total mesorectal excision was performed by dissecting the plane behind the mesorectum. After cancer was removed, the bowel was reconstructed by double-staple technique (sigmoid colon and rectal cancer) or side-to-end anastomosis (cancer of the other segment) except for abdominoperineal resection and Hartmann procedure. An abdominal drain was usually placed in front of the sacrum only in cases of mid- or low rectal cancer.

For hepatectomy, the Pringle maneuver was applied intermittently: on for 10 to 15 minutes, and off for 5 minutes. We utilized the clamp-crushing method for parenchymal dissection along with a bipolar vessel sealing device (BVSD) or ultrasonically activated device (UAD).^[31]^ Major hepatic vessels were closed with a simple ligation, suture ligature, and/or stapler. After tumor removal, hemostasis was achieved by compressing the surgical bed and/or the use of fibrin glue. Biliary leakage was controlled with a suture ligature. Prophylactic abdominal drains were placed selectively.

For laparoscopic colorectal surgery, patients were placed in the modified lithotomy position. Five trocars were used: an umbilical port for the laparoscopic camera and 2 trocars each on the right and left sides. Under pneumoperitoneum (10 mm Hg), the retroperitoneum and mesocolon/mesorectum were divided from a medial-to-lateral approach. After vessel ligations and mobilization of the colorectum, the tumor-bearing segment was pulled out through an extended incision at the umbilicus, and removed. An intracorporal anastomosis was performed by a double-stapling technique, whereas a side-to-end anastomosis was performed extracorporeally.

For laparoscopic hepatectomy, 4 to 6 trocars were placed in the right upper quadrant; some of them were shared with colorectal surgery. The lateral approach with intercostal trocars could be used for lesions in Segment 7 and/or 8.^[32]^ An umbilical tape was passed around the hepatoduodenal ligament for the Pringle maneuver. In a flat or reverse-Trendelenburg position, the liver parenchyma was dissected by the clamp-crushing technique with BVSD/UAD under pneumoperitoneum (12 mm Hg).^[32]^ Major hepatic vessels were divided by ligation and/or a stapler. Bleeding from small vessels and bile leakage were controlled similarly to open hepatectomy. Selective drains were used.

### 2.2 Diagnosis of CRC and metastases

Primary CRC was diagnosed by colonoscopy, contrast enema, and/or computed tomography (CT). Hepatic lesions were detected via preoperative image studies such as contrast-enhanced CT, gadolinium ethoxybenzyl diethylenetriamine pentaacetic acid-enhanced magnetic resonance imaging (MRI), and ultrasound. Intraoperatively, contrast-enhanced intraoperative ultrasonography^[33]^ and indocyanine green (ICG) fluorescence imaging^[34]^ were used to identify metastases.

### 2.3 Follow-up

Adjuvant chemotherapy was recommended, but it depended on patients’ preference and doctor's considerations. Patients were evaluated by a physical examination, measurement of tumor markers, computed tomography scans every 6 months, and colonoscopy annually after curative surgery. Other imaging studies such as MRI and positron emission tomography were additionally performed in cases of rapid increases in tumor marker levels or a new manifestation of symptoms suggesting tumor relapse.

### 2.4 Patient groups

From our database, we searched for consecutive patients diagnosed as having CRC with synchronous liver metastases who underwent simultaneous resections for all lesions in our hospital between January 2011 and March 2019. Among the 76 patients identified, we excluded 4 patients who underwent the first-stage of planned 2-stage hepatectomy.

According to each surgical approach for primary tumor and hepatic lesions, the remaining patients were classified into either the Op-Op (totally open surgery), Lap-Op (laparoscopic colorectal surgery and open hepatectomy), or Lap-Lap group (totally laparoscopic surgery). The Lap-Op and Lap-Lap groups included patients who received conversion to open laparotomy during surgery. Since only 1 patient underwent laparoscopic hepatectomy and open colectomy, the presumptive Op-Lap group was not created. As a consequence, 71 patients were analyzed.

### 2.5 Data collection

The following data were retrieved: gender, age, American Society of Anesthesiologists physical status classification, body mass index, hepatitis B surface antigen, hepatitis C virus antibody, Child-Pugh class, ICG-R15 value, serum carcinoembryonic antigen level before surgery, location and size of primary tumor, and location, number, maximum diameter of hepatic lesions, extrahepatic metastases, and preoperative treatment (chemoradiation therapy, radiotherapy, or chemotherapy).

We reviewed perioperative parameters such as total operative time, Pringle time, order of surgical procedures, other surgical procedures, conversion to open laparotomy, estimated blood loss during surgery, intraoperative transfusion, number of trocars in laparoscopic surgery, number of abdominal drainage tubes, postoperative complications, and hospital stay after surgery. Complications were graded according to the Clavien-Dindo classification.^[35]^

Relapse-free survival (RFS) was defined as the time between the curative-intent surgery and diagnosis of recurrence, and overall survival (OS) as the time between the surgery and death from any cause.

### 2.6 Statistics

Statistical analyses were performed with the JMP 15.1.0 (SAS Institute, Cary, NC). Continuous variables were compared by the Kruskal–Wallis or Mann–Whitney *U* test; the Steel–Dwass test was further used for multiple comparisons. Categorical variables were summarized as percentages and compared by Yates-corrected *χ*^2^ or Fisher exact test where appropriate; the Bonferroni correction was further used for multiple testing. To estimate RFS and OS in patients with liver-limited metastasis who underwent curative resections, the Kaplan–Meier method was applied with the Log-rank test. *P* values <.05 were considered significant in all tests except for multiple testing using the Bonferroni adjustment where the significance level was changed to 0.05/3=0.017.

### 2.7 Ethics

This study was conducted following approval by the ethics committee of the University of Tokyo Hospital (No. 3252-10).

## 3 Results

Among 71 patients (36 men, median age: 63 years), the Op-Op, Lap-Op, and Lap-Lap groups comprised 36, 18, and 17 patients, respectively. Table 1 shows the patients’ characteristics. Rectal cancer was the most frequent primary tumor (45%). Segment 7 was the most frequently metastasized location in the liver (44%); lesions in Segment 8 were significantly fewer as targets for laparoscopic hepatectomy (*P* = .005). The Op-Op and Lap-Op groups included more patients with metastases involving the superior/posterior segments than the Lap-Lap (*P* = .0005). There were no significant intergroup differences in other preoperative factors.

**Table 1:** Patient characteristics.

		Op-Op	Lap-Op	Lap-Lap	
		(n = 36)	(n = 18)	(n = 17)	*P* value
Gender	Male	22 (61%)	8 (44%)	6 (31%)	.31
	Female	14 (39%)	10 (56%)	11 (69%)	
Age (yr)	Median (range)	68 (40–86)	62 (40–78)	64 (49–75)	.64
ASA-PS	1	13 (36%)	9 (50%)	6 (38%)	.96
	2	22 (61%)	9 (50%)	10 (56%)	
	3	1 (3%)	0 (0%)	1 (6%)	
BMI (kg/m^2^)	Median (range)	20.5 (14.9–30.7)	21.5 (16.2–28.8)	22.4 (18.3–28.8)	.22
HBs-Ag	Positive	2 (6%)	0 (0%)	1 (6%)	.92
HCV-Ab	Positive	0 (0%)	0 (0%)	1 (6%)	.77
Child-Pugh	Class A	36 (100%)	18 (100%)	17 (100%)	1.00
ICG-R15^∗^	≤ 15%	34 (94%)	17 (94%)	16 (94%)	.74
	> 15%	2 (6%)	1 (6%)	1 (6%)	
Serum CEA	Normal	7 (19%)	3 (17%)	5 (29%)	.85
	Elevated	29 (81%)	15 (83%)	12 (71%)	
Location of	Right-sided colon	14 (39%)	0 (0%)	4 (24%)	.087
primary tumor	Left-sided colon	8 (22%)	7 (39%)	6 (35%)	
	Rectum	14 (39%)	11 (61%)	7 (41%)	
Size of primary tumor (mm)	Median (range)	40 (12–92)	44 (23–76)	50 (18–85)	.62
Number of hepatic lesions^†^	Median (range)	2 (1–25)	2 (1–12)	1 (1–11)	.25
Maximum size of hepatic lesions (mm)	Median (range)	25 (3–150)	17 (5–90)	15 (7–54)	.054
Location of	Segment 1	3 (8%)	1 (6%)	0 (0%)	.74
hepatic lesions	Segment 2	11 (31%)	2 (11%)	3 (18%)	.42
	Segment 3	11 (31%)	1 (6%)	5 (29%)	.22
	Segment 4a	11 (31%)	7 (39%)	1 (6%)	.16
	Segment 4b	11 (31%)	4 (22%)	2 (12%)	.51
	Segment 5	12 (33%)	3 (17%)	5 (29%)	.62
	Segment 6	12 (33%)	2 (11%)	7 (41%)	.24
	Segment 7	15 (42%)	12 (67%)	4 (24%)	.080
	Segment 8	18 (50%)	7 (39%)	0 (0%)	.005^‡^
	Superior/Posterior Segments^§^	26 (72%)	16 (89%)	4 (24%)	.0005^||^
Extrahepatic metastasis	Present	2 (6%)	1 (6%)	4 (24%)	.27
	Lung	1 (3%)	1 (6%)	1 (6%)	.92
	Peritoneum	1 (3%)	0 (0%)	2 (12%)	.61
	Distant nodes	0 (0%)	0 (0%)	2 (12%)	.29
Preoperative therapy	CRT	2 (6%)	1 (6%)	2 (12%)	.94
	Radiotherapy	0 (0%)	1 (6%)	0 (0%)	.77
	Chemotherapy	6 (17%)	2 (11%)	1 (6%)	.79

ASA-PS = American Society of Anesthesiologists physical status, BMI = body mass index, CEA = carcinoembryonic antigen, CRT = chemoradiation therapy, HBs-Ag = hepatitis B virus surface antigen, HCV-Ab = hepatitis C virus antibody, ICG = indocyanine green, Lap-Lap = laparoscopic colorectal resection and hepatectomy, Lap-Op = laparoscopic colorectal resection and open hepatectomy, Op-Op = open colorectal resection and hepatectomy.

∗ Excluding cases without available data.

† Including cases with diagnosis of pathologically complete response and benign lesions in the liver.

‡ *P* value was .0002 for Op-Op versus Lap-Lap using the Bonferroni correction.

§ Segments 1, 4a, 7, and/or 8.

|| *P* value was .002 for Op-Op versus Lap-Lap and .0001 for Lap-Op versus Lap-Lap using the Bonferroni correction.

As shown in Table 2, the total operative time tended to be longer in the order Lap-lap > Lap-Op > Op-Op (516, 493, and 484 min, respectively). The total Pringle time was shorter in laparoscopic hepatectomy than in open hepatectomy; no portal clamping was used in 65% in the Lap-Lap group. Hepatectomy was preferentially selected as the first procedure in the Op-Op group (89%), whereas colorectal surgery was initially performed in about 80% via laparoscopic approaches (*P* < .0001). Major hepatectomy (resection of ≥3 segments) was performed only via totally open laparotomy. Conversion to laparotomy was required during laparoscopic hepatectomy in 3 patients; the reasons included newly detected tumors located in deep areas of the liver, suspected disseminated lesions on the diaphragm, and technical difficulty. The estimated blood loss was 575 mL in the Op-Op group, approximately twice those of the other groups (*P* = .008).

**Table 2:** Surgical procedures.

		Op-Op	Lap-Op	Lap-Lap	
		(n = 36)	(n = 18)	(n = 17)	*P* value
Total operative time (min)	Median (range)	484 (258–771)	493 (340–605)	516 (253–798)	.74
Total pringle time (min)	Median (range)	57 (0–202)^∗^	49 (15–125)	0 (0–102)	.0005^†^
Order of procedures	Colorectal resection first	4 (11%)	16 (89%)	13 (76%)	<.0001^‡^
	Hepatectomy first	32 (89%)	2 (11%)	4 (24%)	
Colorectal surgery	Right-sided colectomy	15 (42%)	0 (0%)	4 (24%)	.27
	Left-sided colectomy	6 (17%)	6 (33%)	5 (29%)	
	Anterior resection	12 (33%)	11 (61%)	7 (41%)	
	Hartmann procedure	0 (0%)	1 (6%)	0 (0%)	
	Abdominoperineal resection	3 (8%)	0 (0%)	1 (6%)	
Liver surgery	Single nodulectomy	12 (33%)	9 (50%)	9 (53%)	1.00
	Multiple nodulectomies	17 (47%)	9 (50%)	8 (47%)	
	Segmentectomy + multiple nodulectomies	2 (5%)	0 (0%)	0 (0%)	
	Bi-segmentectomy + single nodulectomy	1 (3%)	0 (0%)	0 (0%)	
	Bi-segmentectomy + multiple nodulectomies	1 (3%)	0 (0%)	0 (0%)	
	LH	1 (3%)	0 (0%)	0 (0%)	
	Extended LH + single nodulectomy	1 (3%)	0 (0%)	0 (0%)	
	Extended RH+ single nodulectomy	1 (3%)	0 (0%)	0 (0%)	
	Major hepatectomy	3 (9%)	0 (0%)	0 (0%)	.66
Procedures other than colectomy and hepatectomy^§^		5 (14%)	1 (6%)	2 (12%)	0.86
Conversion to open surgery		N/A	0 (0%)	3 (18%)	0.088
Total number of trocars^||^	Median (range)	N/A	5 (5–5)	8 (5–10)	<.0001
Total number of abdominal drains	Median (range)	2 (0–4)	1 (0–3)	1 (0–4)	.001^¶^
Blood loss (mL)	Median (range)	575 (90–1840)	270 (42–1,155)	290 (15–1460)	.008^#^
Intraoperative transfusion		10 (28%)	1 (6%)	1 (6%)	0.13
Surgical margin	R0	28 (78%)	18 (100%)	17 (100%)	.056
	R1	8 (22%)	0 (0%)	0 (0%)	

Lap-Lap = laparoscopic colorectal resection and hepatectomy, Lap-Op = laparoscopic colorectal resection and open hepatectomy, LH = left hepatectomy, N/A = not available, Op-Op = open colorectal resection and hepatectomy, RH = right hepatectomy.

∗ Excluding one unavailable case.

† *P* value was .001 for Op-Op versus Lap-Lap, and .002 for Lap-Op versus Lap-Lap by the Steel–Dwass test.

‡ *P* value was <.0001 for Op-Op versus Lap-Op, and <.0001 for Op-Op versus Lap-Lap using the Bonferroni correction.

§ Excluding cholecystectomy and stoma creation.

|| Excluding cases of conversion.

¶ *P* value was .004 for Op-Op versus Lap-Op, and .017 for Op-Op versus Lap-Lap by the Steel–Dwass test.

# *P* value was .006 for Op-Op versus Lap-Op by the Steel–Dwass test.

Positive surgical margins were reported in the liver specimens in 22% of the Op-Op group, whereas complete resection was achieved in all Lap-Op and Lap-Lap groups (*P* = .056). Seven of 180 resected hepatic lesions were histologically diagnosed as primary benign hepatic tumors, 11 as other benign lesions, 1 as a hepatocellular carcinoma, and 1 as a primary cholangiocarcinoma. In addition, 3 lesions turned out to be scars without viable cancer cells after chemotherapy.

The overall complication rate of grade 2 was similar among the groups. The median hospital stays for the Lap-Lap and Lap-Op groups were shorter than that for the Op-Op (17, 15 vs 19 days, *P* = .033, Table 3).

**Table 3:** Postoperative outcomes.

	Op-Op	Lap-Op	Lap-Lap	
	(n = 36)	(n = 18)	(n = 17)	*P* value
Postoperative complications (grade 2-)				
Overall	15 (42%)	4 (22%)	5 (29%)	.52
Bile leak	0 (0%)	0 (0%)	0 (0%)	1.00
Anastomotic leak	0 (0%)	0 (0%)	0 (0%)	1.00
Bleeding	2 (6%)	0 (0%)	0 (0%)	.89
Bowel obstruction	3 (9%)	1 (5%)	0 (0%)	.76
Trocar site hernia	0 (0%)	0 (0%)	2 (12%)	.29
Surgical site infection	8 (22%)	2 (11%)	1 (6%)	.50
Nonsurgical site infection	1 (3%)	0 (0%)	1 (6%)	.89
Cardiovascular	0 (0%)	0 (0%)	0 (0%)	1.00
Pulmonary	0 (0%)	1 (5%)	0 (0%)	.77
Renal / urinary	0 (0%)	0 (0%)	0 (0%)	1.00
Others	2 (6%)	1 (5%)	1 (6%)	.74
Hospital stay after surgery (d)				
Median (range)	19 (12–53)	15 (10–71)	17 (8–38)	.033^∗^

Lap-Lap = laparoscopic colorectal resection and hepatectomy, Lap-Op = laparoscopic colorectal resection and open hepatectomy, Op-Op = open colorectal resection and hepatectomy.

∗ *P* value was .0495 for Op-Op versus Lap-Lap by the Steel–Dwass test.

We compared the long-term outcomes of 61 patients with liver-limited metastasis who underwent R0/R1 resections (Table 4). The implementation rate of adjuvant chemotherapy was similar among the groups. The median follow-up period was 57 months for the Op-Op group, 35 months for Lap-Op, and 48 months for Lap-Lap (*P* = .15). The postoperative survivals were similar.

**Table 4:** Long-term outcomes in patients with liver-limited metastases undergoing curative-intent surgery.

		Op-Op	Lap-Op	Lap-Lap	
		(n = 33)	(n = 17)	(n = 11)	*P* value
Adjuvant chemotherapy	None	12 (36%)	6 (35%)	4 (36%)	.98
	5-FU-based	6 (18%)	3 (18%)	2 (18%)	
	5-FU + oxaliplatin	15 (46%)	7 (41%)	5 (46%)	
	5-FU + irinotecan	0 (0%)	1 (6%)	0 (0%)	
Follow-up period (mo)	Median (range)	57 (18–111)	35 (12–87)	48 (16–93)	.15
Relapse-free survival	3-yr survival rate	23%	29%	53%	.40
Overall survival	3-yr survival rate	78%	79%	100%	.37

5-FU = 5-fluorouracil, Lap-Lap = laparoscopic colorectal resection and hepatectomy, Lap-Op = laparoscopic colorectal resection and open hepatectomy, Op-Op = open colorectal resection and hepatectomy.

## 4 Discussion

Previous RCTs independently demonstrated advantages of the laparoscopic approach such as reduced intraoperative bleeding and shorter postoperative recovery in colorectal or hepatic resection.^[10–17,21]^ A multicenter study and systematic reviews found that simultaneous resections of primary CRC and hepatic metastases via a laparoscopic approach seem to provide the aforementioned benefits,^[22,23,26]^ but they were not compared with open surgery or a combination of open and laparoscopic resections Some small studies have reported totally laparoscopic resections of primary CRC and liver metastases in comparison with the counterpart patients undergoing open surgery.^[26,29]^ More recently, the comparative surgical outcomes of combined resections for primary CRC and liver metastases between open and laparoscopic/robotic methods were reported; however, 80% received chemotherapy before surgery,^[27]^ whereas the implementation rate of preoperative chemotherapy was approximately one-third in other institutes where 1-stage minimally invasive surgery for colorectal liver metastasis was performed.^[36]^ Tranchart et al^[28]^ compared 89 patients undergoing laparoscopic simultaneous resections of CRC and liver metastases with 89 treated by laparotomy using propensity score adjustment; the subjects included old cases dating back to 1997. We analyzed a recent cohort comprising a close to real-world proportion of patients receiving preoperative therapies. Notably, this is the first comparison of simultaneous laparoscopic resections of CRC and liver metastases with those via open surgery and a mixed approach.

In previous RCTs, intraoperative blood loss was lower in laparoscopic colorectal surgery than open procedures.^[10–17]^ Consistently, the use of laparoscopy reduced the volume of blood loss by half in the current study. In contrast, longer operative times were reported for laparoscopic colorectal surgery,^[10–17]^ whereas laparoscopic hepatectomy required a similar operative time to open hepatectomy in the OSLO-COMET trial.^[21]^ Conversely, a propensity-score-matched study on 1-stage resections of CRC and liver metastases reported that the laparoscopic approach made operative times shorter for colorectal resection and longer for hepatic resection.^[28]^ In our study, operative times tended to be longer in the order Lap-Lap > Lap-Op > Op-Op.

The OSLO-COMET trial reported a reduced rate of severe morbidities after hepatectomy via laparoscopic approach.^[21]^ In contrast, laparoscopic colorectal surgery showed similar complication rates to open surgery in most trials.^[11–17]^ We did not observe differences in overall complication rates among the groups similarly to a previous study on 1-stage resections of colorectal liver metastases.^[28]^ The length of hospital stay was more than 2 weeks probably because the national health insurance covers a fixed rate of hospitalization costs independently of its length in Japan.

When open hepatectomy is planned, laparoscopic resection of a right-sided colon cancer appears to have little merit; the incisions can be shared for the surgical procedures, which provides a better surgical view and shortens the operative time. This is the main reason why the Lap-Op group did not include a case of right-sided colon cancer.

Lesions involving superior/posterior segments were relatively fewer in laparoscopic hepatectomy than in open hepatectomy; Segments 1 and 8 were exclusively resected by open hepatectomy. They are more difficult to resect laparoscopically, requiring a longer operative time and resulting in greater bleeding than the others.^[32,37]^ Further progress in laparoscopic techniques will improve the safety of hepatectomy for these technically challenging regions, which may extend the indications of the totally laparoscopic approach for colorectal liver metastases.

There used to be substantial concern that portal clamping may cause bowel edema and compromise the healing of the intestinal anastomosis.^[6]^ To avoid the risk of anastomotic complications, hepatectomy was more frequently performed first in the Op-Op group. In the Lap-Op group, colorectal surgery tended to be performed first to obtain a stable pneumoperitoneum. In laparoscopic hepatectomy, low venous pressure achieved by pneumoperitoneum may considerably help reduce bleeding. In fact, the Pringle maneuver was not used without increasing blood loss in many patients in the Lap-Lap group. We do not consider “liver-and-colorectum” to necessarily be the desirable order for patients undergoing 1-stage resections laparoscopically.

This retrospective study has limitations. Several patient characteristics were unbalanced since the treatment choice was not randomized. Due to the small number of patients, we could not adjust these biases. Only the Op-Op group included major hepatectomy, which would also adversely affect surgical outcomes. Whether laparoscopic major hepatectomy should be performed simultaneously or separately from colorectal surgery remains to be investigated. The prognoses of patients in each group did not differ, but the shorter follow-up period in the laparoscopic groups might have influenced the outcomes.

To conclude, laparoscopic surgery for colorectal liver metastases in simultaneous resections provided advantages over open surgery in terms of intraoperative blood loss and postoperative hospital stay in selected patients. Further studies are warranted to verify the benefits provided by a laparoscopic approach in these patients.

## Acknowledgments

The authors thank for Medical English Service (Kyoto, Japan) for editing our manuscript.

## Author contributions

**Conceptualization:** Hiroaki Nozawa, Takeaki Ishizawa.

**Data curation:** Hiroaki Nozawa, Hiroaki Ishii, Hirofumi Sonoda, Shigenobu Emoto, Koji Murono, Kazuhito Sasaki, Kazushige Kawai, Nobuhisa Akamatsu, Junichi Kaneko, Junichi Arita, Kiyoshi Hasegawa.

**Formal analysis:** Hiroaki Nozawa, Takeaki Ishizawa, Hideo Yasunaga.

**Funding acquisition:** Hiroaki Nozawa, Hirofumi Sonoda, Shigenobu Emoto.

**Investigation:** Hiroaki Nozawa, Takeaki Ishizawa, Soichiro Ishihara.

**Methodology:** Hiroaki Nozawa, Takeaki Ishizawa, Soichiro Ishihara.

**Project administration:** Hiroaki Nozawa, Soichiro Ishihara.

**Resources:** Hiroaki Nozawa.

**Software:** Hideo Yasunaga.

**Supervision:** Kiyoshi Hasegawa, Soichiro Ishihara.

**Validation:** Takeaki Ishizawa.

**Visualization:** Hiroaki Nozawa.

**Writing – original draft:** Hiroaki Nozawa, Takeaki Ishizawa.

**Writing – review & editing:** Hideo Yasunaga, Hiroaki Ishii, Hirofumi Sonoda, Shigenobu Emoto, Koji Murono, Kazuhito Sasaki, Kazushige Kawai, Nobuhisa Akamatsu, Junichi Kaneko, Junichi Arita, Kiyoshi Hasegawa, Soichiro Ishihara.
